# 81. Impact of Early Post-Transplant Multidrug-Resistant Organism Detection Among Renal Transplant Recipients, 2005–2021

**DOI:** 10.1093/ofid/ofac492.006

**Published:** 2022-12-15

**Authors:** Ahmed Babiker, Geeta Karadkhele, Chad Robichaux, Alex M Page, Sarah W Satola, Colleen S Kraft, Christian P Larsen, Stephanie M Pouch, Michael H Woodworh

**Affiliations:** Emory University School of Medicine, Atlanta, GA; Emory University School of Medicine, Atlanta, Georgia, Atlanta, Georgia; Emory University, Atlanta, Georgia; Emory University School of Medicine, Atlanta, GA; Emory University School of Medicine, Atlanta, GA; Emory University School of Medicine, Atlanta, GA; Emory University School of Medicine, Atlanta, GA; Emory University School of Medicine, Atlanta, GA; Emory University School of Medicine, Atlanta, GA

## Abstract

**Background:**

Understanding the impact of multi-drug resistant organism (MDRO) acquisition on renal transplant recipients (RTR) mortality and allograft function is paramount to mitigating deleterious outcomes. Prior studies have been limited by lack of control groups and sample sizes. We aimed to the assess whether the detection of an MDRO or a susceptible organism during the early post-transplant period was associated with increased mortality and allograft failure among RTRs.

**Methods:**

We performed a retrospective cohort study of RTRs at the Emory University Transplant Center between 2005–2022. Early post-transplant culture positivity was defined as a positive culture within 30 days of renal transplant. The primary outcome was a combined composite of one year- allograft loss and/or mortality following renal transplant. A Kaplan–Meier survival analysis was performed, and differences between survival curves for RTRs with an early post-transplant positive culture (stratified by susceptibility status) and negative control RTRs were assessed using the log-rank test. Multivariable cox proportional hazard and a competing risk analysis were performed.

**Results:**

Among 3,233 RTRs, 259 (8%) had a susceptible organism detected and 35 (1%) had an MDRO detected (Figure 1). Demographic and microbiology characteristics are summarized in Table 1 **& 2**. One hundred and forty-nine (5%) RTRs experienced the composite outcome, this was experienced more frequently among RTRs with an MDRO detected (14%, 5/35) compared to RTRs with a susceptible organism defected (8%, 21/259) and negative controls (4%, 123/2,939) (Table 3). Significant difference between time from transplantation to the composite outcome when comparing negative controls, MDRO and susceptible organisms RTRs was observed (log rank *p* < 0.001) (Figure 2). Early post-transplant culture positivity (aHR 1.98 [1.30, 3.04]) and MDRO detection (aHR: 3.20 [1.30, 7.84]) were significantly associated with the composite outcome (Table 4).

**Conclusion:**

MDRO as well as susceptible organism acquisition during the early post-transplant period was associated with increased mortality and allograft loss highlighting the need for increased infection prevention efforts within this vulnerable population.

**Disclosures:**

**All Authors**: No reported disclosures.

**Figure 1.**
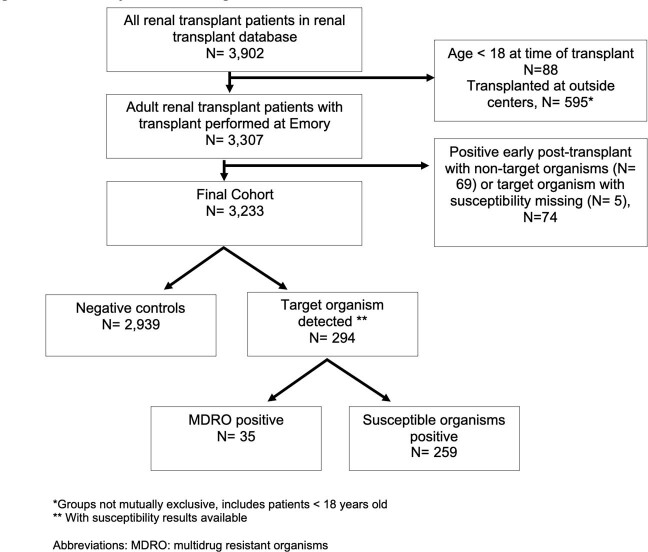
Study Flow Diagram

**Table 1.**
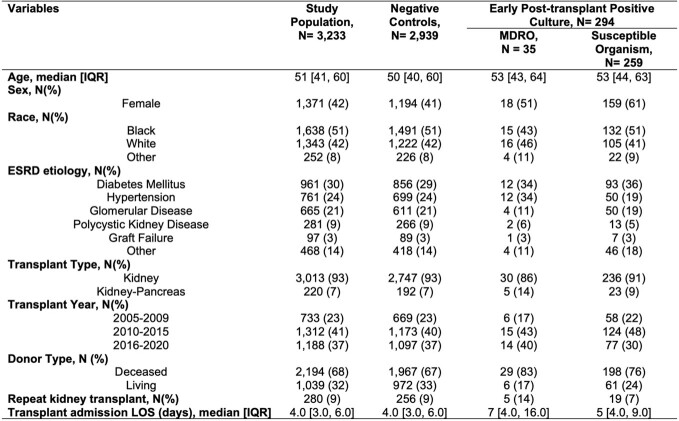
Demographic and Clinical Characteristics of Renal Transplant Recipients by Early Post-Transplant Culture Results Status (N= 3,233)

Abbreviations: IQR: interquartile range, LOS: length of stay, ESRD: end-stage renal disease, MDRO: multidrug-resistant organism

**Table 2.**
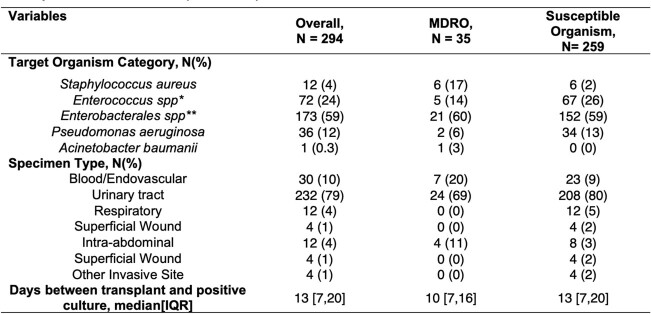
Microbiological Characteristics of Renal Transplant Recipients with Positive Early Post-Transplant Cultures (N=294)

* Enterococcus spp group includes: Enterococcus faecalis (n= 63), Enterococcus faecium (n=9)

** Enterobacterales spp group includes: Citrobacter freundii complex (n= 8), Enterobacter cloacae complex (n=7), Escherichia coli (n=97), Klebsiella aerogenes (n=3), Klebsiella oxytoca (n=10), Klebsiella pneumoniae (n=31), Proteus mirabilis (n=9), Proteus vulgaris (n=1), Providencia rettgeri (n=1), Serratia marcescens (n=6)

Abbreviations: IQR: interquartile range, MDRO: multidrug-resistant organism

**Table 3.**
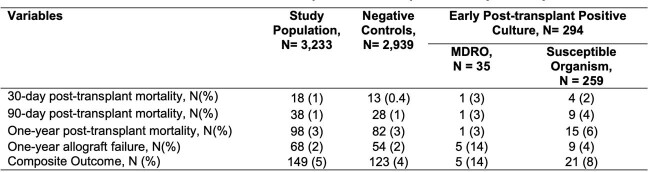
Outcomes of Renal Transplant Recipients by Early Post-Transplant Positivity Status

Abbreviations: IQR: interquartile range, MDRO: multidrug-resistant organism

**Table 4.**
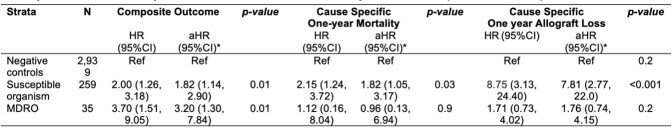
Results of Cox Proportional and Cause-Specific Hazards Model for Primary Outcome by Early Post-transplant Culture Positivity Status (N= 3,233)

Abbreviations: aHR: adjusted hazard ratio, HR: hazard ratio, MDRO: multidrug-resistant organism

*Adjusted for age, year of transplant, repeat transplant status and multiple organ transplantation status

**Figure 2:**
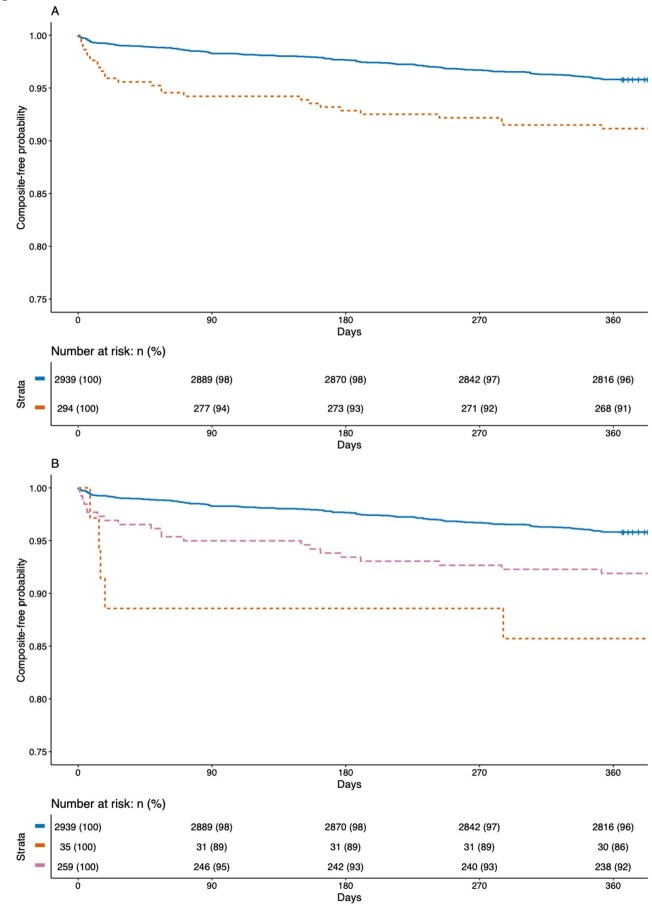
Kaplan–Meier analysis of composite outcome comparing renal transplant recipients with a positive early post-transplant culture (orange) and negative controls (blue) (A) and comparing renal transplant recipients with anmultidrug-resistant organism detected on early post-transplant culture (orange) a susceptible organism detected on post-transplant culture (pink) and negative controls (blue) (B). Time is measured from transplant until event. Log-rank p < 0.001.

**Figure 3:**
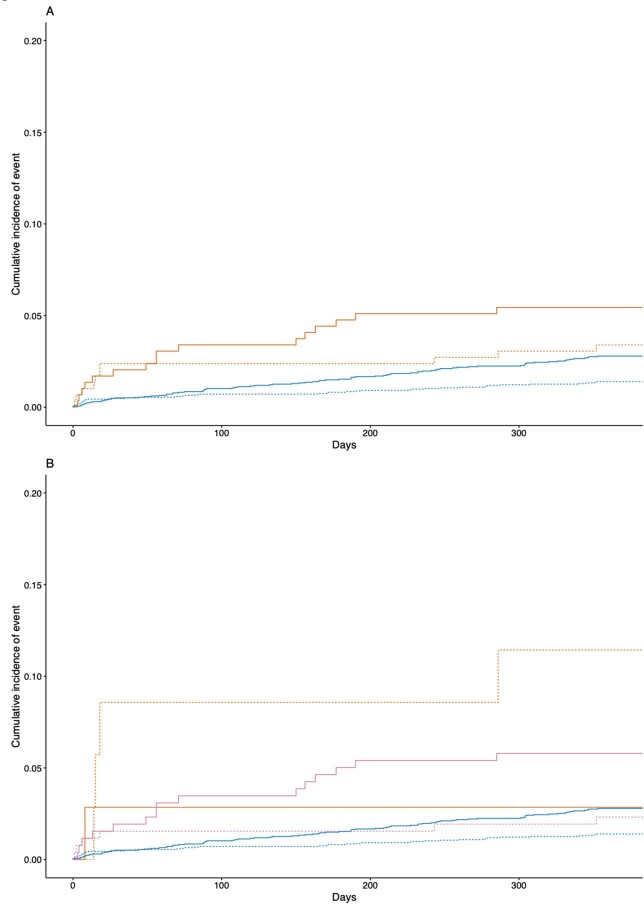
Cumulative incidence curves for one-year mortality (solid line) and one-year allograft loss (dashed line) comparing renal transplant recipients with a positive early post-transplant culture (orange) and negative controls (blue) (A) and comparing renal transplant recipients with an multidrug-resistant organism detected on early post-transplant culture (orange), a susceptible organism detected on post-transplant culture (pink) and negative controls (blue) (B). Time is measured from transplant until event.

